# SiO and CH_3_OH mega-masers in NGC 1068

**DOI:** 10.1038/ncomms6449

**Published:** 2014-11-11

**Authors:** Junzhi Wang, Jiangshui Zhang, Yu Gao, Zhi-Yu Zhang, Di Li, Min Fang, Yong Shi

**Affiliations:** 1 Shanghai Astronomical Observatory, Chinese Academy of Sciences, 80 Nandan Road, Shanghai 200030, China; 2 School of Astronomy and Space Science, Nanjing University, Nanjing 210093, China; 3 Key Laboratory of Radio Astronomy, Chinese Academy of Sciences, Nanjing 210008, China; 4 Center For Astrophysics, GuangZhou University, GuangZhou 510006, China; 5 Purple Mountain Observatory, Chinese Academy of Sciences, 2 West Beijing Road, Nanjing 210008, China; 6 Institute for Astronomy, University of Edinburgh, Royal Observatory, Blackford Hill, Edinburgh EH9 3HJ, UK; 7 National Astronomical Observatories, Chinese Academy of Sciences, 20A Datun Road, Chaoyang District, Beijing 100012, China; 8 Space Science Institute, Boulder, Colorado 80301, USA; 9 Key Laboratory of Modern Astronomy and Astrophysics (Nanjing University), Ministry of Education, Nanjing 210093, China

## Abstract

Maser is an acronym for microwave amplification by stimulated emission of radiation; in astronomy mega-masers are masers in galaxies that are ≥10^6^ times more luminous than typical galactic maser sources. Observational studies of mega-masers can help us to understand their origins and characteristics. More importantly, mega-masers can be used as diagnostic tracers to probe the physical properties of their parent galaxies. Since the late 1970s, only three types of molecules have been found to form mega-masers: H_2_O, OH and H_2_CO. Here we report the detection of both SiO and CH_3_OH mega-masers near the centre of Seyfert 2 galaxy NGC 1068 at millimetre wavelengths, obtained using the IRAM 30-m telescope. We argue that the SiO mega-maser originated from the nuclear disk and the CH_3_OH mega-maser originated from shock fronts. High-resolution observations in the future will enable us to investigate AGN feedback and determine the masses of central supermassive black holes in such galaxies.

## Abstract (web summary)

Astrophysical masers are interstellar sources of stimulated spectral line emission, with most SiO masers in the Milky Way existing around evolved stars. Here, Wang *et al.* report detections of millimetre SiO and Class I CH_3_OH mega-masers in NGC 1068.

## Graphical abstract

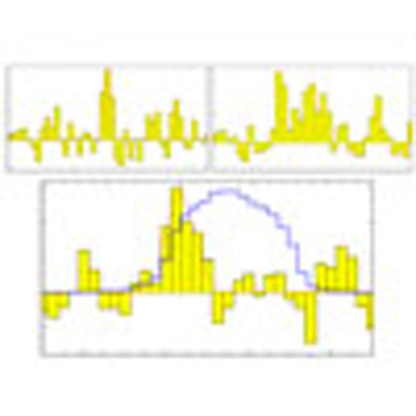

Most of the SiO (silicon monoxide) masers in the Milky Way are known to occur around evolved stars and are collisionally pumped^1^, which reveal circumstellar envelope dynamics at high resolution^2^. A relatively low-level extragalactic SiO maser has been detected in the Large Magellanic Cloud with the same properties as SiO masers from the Milky Way sources^3^. Similarly, CH_3_OH (methanol) masers trace massive star-forming regions^1^, where Class I masers are pumped by collision and Class II masers are pumped by radiation^4^. Three species (H_2_O (ref. 5), OH (ref. 6) and H_2_CO (ref. 7)) of extremely luminous masers, which are known as mega-masers^8^, have been found in galaxies. The 22.235 GHz H_2_O mega-maser provides a powerful probe of the accretion disk and supermassive black holes (SMBHs) in active galactic nuclei (AGNs), and enables the accurate determination of the angular-diameter distance of maser host galaxies, independent of standard candle arguments^8^,^9^. However, OH mega-masers are excellent tracers of heavily dust-embedded starbursts in the inner 100 pc of local ultra-luminous IR galaxies^8^,^10^. Numerous studies have been conducted to search for new mega-maser molecules for more than 30 years, without success^11^,^12^,^13^. A Class I extragalactic CH_3_OH maser has been recently found in NGC 253 that is 17 times more luminous than similar emissions near the galactic centre^14^ and is still not a mega-maser.

In this paper, we report the detection of millimetre SiO and CH_3_OH mega-masers at approximately the 5 *σ* level in the Seyfert 2 galaxy NGC 1068 using the IRAM 30-m telescope. Our results increase the number of known mega-maser molecules from three to five and may present new opportunities for studying AGN with the current powerful millimetre facilities, such as the Atacama Large Millimeter/submillimeter Array (ALMA).

## Results

**Figure 1:**
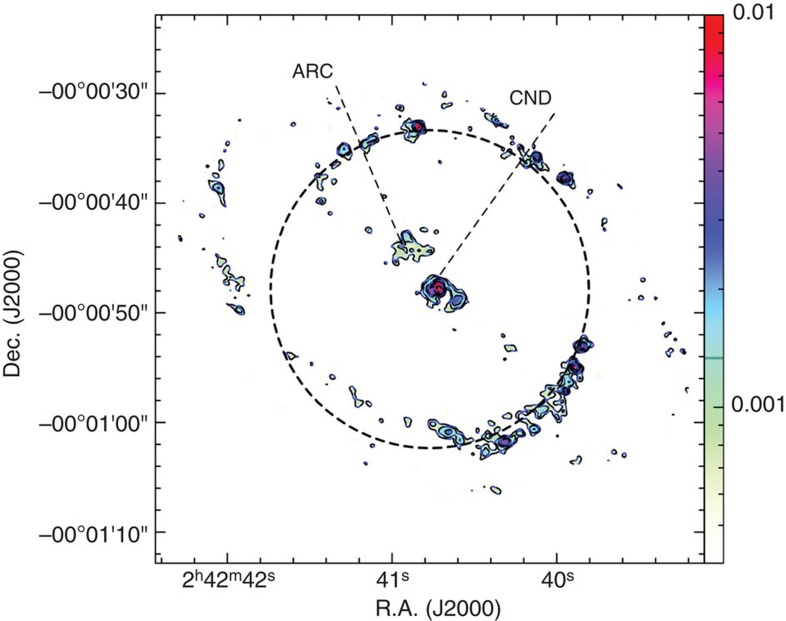
Sub-millimetre dust emission in NGC 1068. Three hundred and forty-nine gigahertz dust continuum emission from ALMA observation^16^. The ‘CND’ is circumnuclear disk and ‘ARC’ is a ‘bow-shock’ arc defined by 349 GHz dust emission obtained by ALMA observation^16^. The dashed circle shows the beam of IRAM 30-m, while the colour scale is for the flux in the unit of Jy per beam shown with the colour bar in the right.

**Figure 2:**
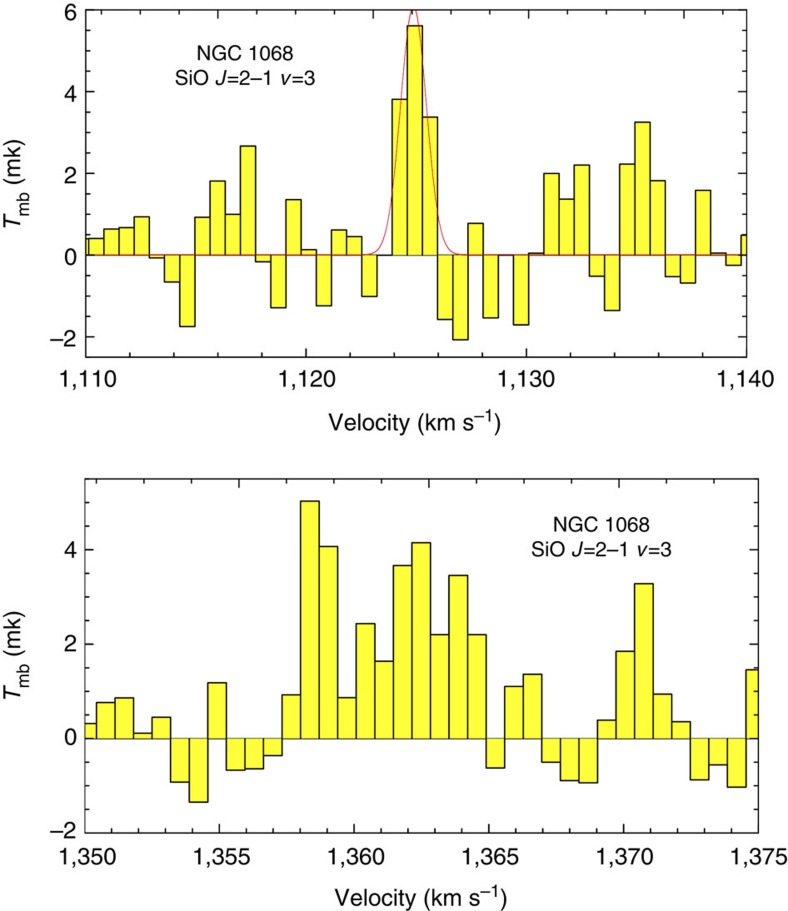
SiO maser emissions detected in NGC 1068. Top: SiO *J*=2−1 (*υ*=3) maser emission at the velocity of 1,125 km s^−1^ in NGC 1068 detected with IRAM 30-m telescope, the noise level is about 0.93 mK at this velocity resolution (~0.69 km s^−1^), The *x* axis is the radio-defined velocity of SiO *J*=2−1 (*υ*=3), while the *y* axis is the main beam brightness temperature. The red line is the Gaussian fitting. Bottom: the same as that in the top but at the velocity of 1,362 km s^−1^.

**Figure 3:**
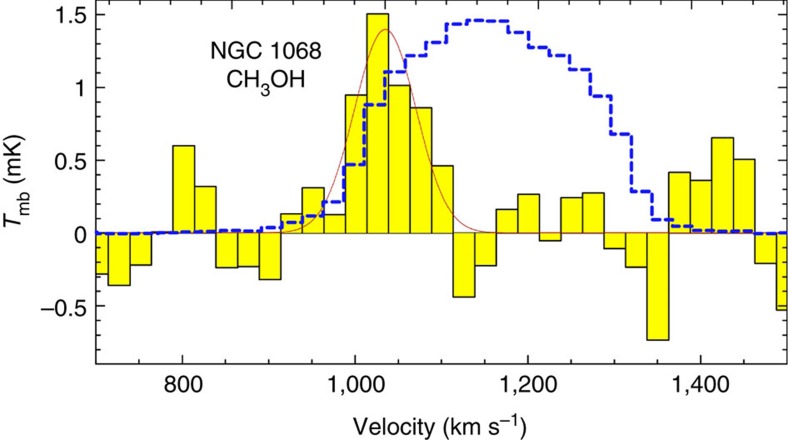
CH_3_OH maser emission detected in NGC 1068. CH_3_OH 5_−1_-4_0_*E* spectrum in NGC 1068 (black line and filled yellow histogram), smoothed to a velocity resolution of 24.9 km s^−1^, which gave the noise level of 0.37 mK. The *x* axis is the radio-defined velocity of CH_3_OH 5_−1_-4_0_*E*, while the *y* axis is the main beam brightness temperature. The blue dashed line represents the simultaneously obtained HCN 1-0 spectrum, which has been scaled down by a factor of 50, while the red line is the Gaussian fitting of the CH_3_OH 5_−1_-4_0_*E* spectrum.

In Dec 2011, we used the IRAM 30-m telescope to observe NGC 1068, which hosts a circumnuclear gas disk (CND) within its inner 5′′ and two spiral arms that are rich in molecular^15^ and dust emissions (see Fig. 1)^16^. We detected emissions of SiO *J*=2−1 (*υ*=3) at 85.038 GHz and CH_3_OH 5_−1_-4_0_*E* at 84.521 GHz as mega-masers at the ~5 *σ* level. The SiO *J*=2−1 (*υ*=3) emissions were detected at the velocity of 1,125 km s^−1^ with a FWHM (full width at half maximum) line width of only 1.5 km s^−1^ and the velocity of 1,362 km s^−1^ with a full width of ~8 km s^−1^ (see Fig. 2), whereas the CH_3_OH emission was detected at the velocity of 1,035 km s^−1^ with a FWHM width of ~84 km s^−1^ (see Fig. 3).

## Discussion

The only SiO *J*=2−1 (*υ*=3) line that was detected in the Milky Way was a maser instead of a thermal emission^17^, and the detected SiO lines in NGC 1068 had much narrower line widths than the thermal lines (HCN 1-0, HCO^+^ 1-0, etc.); thus it was reasonable to assign the SiO line in NGC 1068 to a maser emission. However, the CH_3_OH 5_−1_-4_0_*E* line in the galactic sources could correspond to a maser emission, such as in DR21(OH) (ref. 18), or to thermal emissions, as in Orion^19^ and W3(H_2_O) (ref. 20). Thermal emissions of four CH_3_OH lines (2_−1_-1_−1_*E* at 96.739 GHz, 2_1_-1_0_*A*^+^ at 96.741 GHz, 2_0_-1_0_*E* at 96.744 GHz and 2_1_-1_1_*E* at 96.755 GHz) were detected as one line in an ALMA observation^21^ because of the line broadening, which resulted in a flux of 1.05±0.03 Jy km s^−1^ in the nuclear region of NGC 1068. Each of the four CH_3_OH lines had a similar or an even higher Einstein *A* coefficient and a lower excitation condition than that of the 5_−1_-4_0_*E* transition^19^. The thermal flux of the 5_−1_-4_0_*E* line was <1/4 of that detected at 96.7 GHz in the nuclear region (that is, 0.26 Jy km s^−1^), which was to be expected; however, the detected CH_3_OH 5_−1_-4_0_*E* line flux was 0.53±0.1 Jy km s^−1^ and could not be explained as a pure thermal emission. Another piece of evidence, indicating that the 5_−1_-4_0_*E* line was not a pure thermal emission, was that the line was much narrower than the thermal lines (such as HCN 1-0), and line centre was shifted with respect to the thermal lines (see Fig. 3). Thus, we suggest that the detected CH_3_OH 5_−1_-4_0_*E* line in NGC 1068 originated from a maser emission instead of a thermal emission.

The only source that has been reported for SiO *J*=2−1 (*υ*=3) emissions is *χ* Cyg at a distance of 110 pc (ref. 22), with 0.23 and 0.37 K km s^−1^ at two epochs^17^, which yields an isotropic luminosity of ~0.2 Jy km s^−1^ kpc^2^. The isotropic luminosity of two velocity components of SiO *J*=2−1 (*υ*=3) in NGC 1068 were 7.7±1.5 × 10^6^ and 2.6±0.24 × 10^7^ Jy km s^−1^ kpc^2^ at velocities of 1,125 and 1,362 km s^−1^, respectively, both of which match the criterion of a mega-maser as being ≥10^6^ times more luminous than typical galactic maser sources^8^. Using the Orion molecular cloud(OMC)-2 as a typical galactic maser source of CH_3_OH 5_−1_-4_0_*E* (ref. 23), with a flux of ~200 Jy km s^−1^, yields an isotropic luminosity of 34.3 Jy km s^−1^ kpc^2^, compared with 1.1±.02 × 10^8^ Jy km s^−1^ kpc^2^ for NGC 1068; thus this source can be considered to be a mega-maser of CH_3_OH 5^−1^-4_0_*E*.

Our detected SiO maser exhibits extraordinary luminosity and has different properties from those of the Milky Way sources. The extremely narrow width of the detected SiO *J*=2−1 (*υ*=3) line shows that it is impossible that the SiO emissions in NGC 1068 originated from more than hundreds of millions of the S-type Mira variable stars that have been detected in the Milky Way^17^. SiO masers in the Milky Way have been found with *υ*=1, 2 and 3 at multiple *J* transitions, whereas line intensities normally decrease with the increments in *υ* for the same *J* transition^17^. The SiO *J*=2−1 (*υ*=1 and 2) transitions were also in the frequency range of our observation. We did not detect SiO *J*=2−1 (*υ*=2) at similar noise levels to that of SiO *J*=2−1 (*υ*=3), whereas we marginally detected SiO *J*=2−1 (*υ*=1) at similar velocities (see Supplementary Fig. 3). This result shows that the excitation condition of the SiO molecules in NGC 1068 is different from that of the Milky Way sources and could be very close to that of the AGN, in which the gas temperatures are sufficiently high because the upper level energy of SiO *J*=2−1 (*υ*=3) is equivalent to ~3,660 K above the ground state. Molecular gas with a high column density of an inverted population of SiO (*υ*=3, *J*=2 and *υ*=3, *J*=1) is required to produce the detected mega-maser emission. The enhanced SiO abundance in the nuclear region^24^ supplies SiO molecules for amplifying the emission under special excitation conditions.

Extragalactic Class II CH_3_OH masers have been detected in Large Magellanic Cloud^11^,^25^,^26^,^27^,^28^ and M 31 (ref. 29) with similar properties to those of star-forming regions in the Milky Way. Numerous studies have been conducted on searching for Class II CH_3_OH mega-masers over the past decades, without success^12^,^13^. Recently, a Class I extragalactic CH_3_OH maser was detected in NGC 253 at 36.2 GHz and exhibited an isotropic luminosity to more than one order of magnitude higher than that in the centre of the Milky Way^14^. Class II CH_3_OH masers are radiatively pumped and believed to be signposts of an early phase of massive star formation^29^, whereas Class I CH_3_OH masers originate from collisional excitation^4^. Both the CH_3_OH masers in NGC 253 at 36.2 GHz (ref. 14) and that we detected in NGC 1068 are hard to explain in terms of simple collections of thousands to millions of masers in massive star-forming regions and are Class I instead of Class II. It may be easier to increase the amplification length from collisional excitation than that from radiative pumping and thus easier to form mega-masers. Class I CH_3_OH masers at 84.5 and 36.2 GHz can be used together to determine the excitation conditions for the CH_3_OH molecule and further study the gas properties near AGN, such as NGC 1068, and nuclear starbursts, such as NGC 253.

Both velocities of SiO emissions correspond to the velocities of the nuclear masers for the S1 component of the H_2_O mega-maser; the 1,125 km s^−1^ component is near the systemic velocity that is located toward the nuclear continuum peak of NGC 1068 for the H_2_O mega-maser in the nuclear disk, and the 1,362 km s^−1^ component is associated with the red component of the H_2_O nuclear maser that is located northwest of the continuum peak^30^. However, the velocity of CH_3_OH emission agrees well with the velocity of the C component of jet masers of the H_2_O mega-maser^30^. The different velocities and line widths of SiO and CH_3_OH lines show that these masers originated from different regions under different excitation conditions. It is likely that the SiO masers originated from the nuclear disk, whereas the CH_3_OH emissions originated from the shock front region where the nuclear jets (or outflows) interact with circumnuclear molecular clouds, which could be confirmed by future ALMA observation at the resolution of ~0.1′′.

Thus, NGC 1068 is a unique case that exhibits luminous maser emissions of different molecules: H_2_O, SiO and CH_3_OH. Detailed studies of these mega-masers provide the opportunity to investigate the existence of SiO masers that require a pumping condition and the production of SiO molecules, which could result from AGN feedback in the nuclear region by X-ray radiation and/or shocks^16^,^24^. Maser spots near the nuclear regions can be observed using ALMA and located to a precision of several milliarcseconds, as has been achieved for H_2_O masers in NGC 1068 by VLA observations at A configurations with a beam size of ~0.08 arcsecond (ref. 30). Such observations can be used to study feedback from AGN in NGC 1068 to the innermost region of the molecular disk, whereas such studies are impossible for thermal SiO emission because of the absolute resolution and a limited brightness temperature. As CH_3_OH emissions are likely from the shock front region, a combination of SiO and CH_3_OH maser emissions can be used to study AGN feedback at sub-pc to ~100 pc scales.

SiO mega-masers in nearby type II AGNs near the central SMBHs can be used as probes of AGN feedback and to directly measure the masses of the central SMBHs, as has been achieved using H_2_O mega-masers in nearby type II AGNs with <20 sources because of the limited sensitivity of current VLBI facilities^31^. Adopting the technique that has been used for H_2_O with VLA observation^30^, the structure of maser spots can be determined to ~2 milliarcseconds at the highest ALMA resolution of ~0.05′′. This structure corresponds to ~1 pc at a distance of 100 Mpc, which is primarily dominated by the gravity of the central SMBH.

## Methods

### Observation and data reduction

The observations were performed at the end of Dec 2011 using the IRAM 30-m telescope over 3 days under excellent weather conditions. The Eight MIxer Receiver (EMIR), the FTS backend and standard wobbler switching mode with ±120′′ offset at 0.5-Hz beam throwing, were used. The FTS at the wide band mode provided 8-GHz frequency coverage at 3-mm band and 195 kHz channel width spacing, which corresponds to ~0.69 km s^−1^ at 85 GHz. We determined whether the signal was from the sky frequency instead of from radio-frequency interference at the IF frequency or from the backend by separating the observations into two sub-configurations: on the first day, we set the H^13^CN 1-0 (86.340176 GHz) at the centre of the lower outer part of the EMIR, while we set the H^13^CO^+^ 1-0 (86.754288 GHz) at the centre of lower outer part of the EMIR for the other two days. The total observation time was ~27 h, including tuning, pointing, focusing and the overhead of wobbler switching, which resulted in an effective on+off source time of ~15 h.

The data were reduced with the CLASS package of GILDAS. We checked the data quality of each spectrum, which was read out every 12 min. All of the spectra exhibited good baseline and were thus all used in scientific analysis. The three SiO *j*=2−1 (*υ*=1, 2, 3) maser lines and the SiO *J*=2−1 (*υ*=0) thermal line were within the frequency range of our observation (~84–92 GHz). The typical system temperature during the observations was ~110 K. We averaged all of the spectra with the weight of the on-source time for final analysis, and we obtained averaged spectra for the two frequency tunings. Supplementary Figure 1 shows the final averaged spectrum using all of the data with an 8-GHz wide frequency coverage, which was only subtracted by a first-order baseline fitting and smoothed to a velocity resolution of ~24.3 km s^−1^.

### Identification of SiO emission features

The SiO *J*=2−1 (*υ*=0, 1, 2, 3) lines were within the frequency range of our observation. Our data for the thermal line (*υ*=0) were consistent with previous results that were detected using the IRAM 30-m telescope^32^,^33^ and the IRAM PdBI^24^; no reports of observations of masers lines could be found in the literature. Among the three transitions, we found that the strongest emission feature of the peak brightness temperature corresponded to SiO *J*=2−1 (*υ*=3) at a velocity of 1,125 km s^−1^, with the velocity-integrated flux of ~5 *σ*, which was obtained using a single Gaussian profile fitting. We confirmed that the signal corresponded to the frequency of SiO *J*=2−1 (*υ*=3) and was not a radio-frequency interference at the IF frequency or a feature from a bad bandpass of the backend, by comparing the spectra from two different frequency tunings (see Supplementary Fig. 2): a SiO *J*=2−1 (*υ*=3) emission was detected at the velocity of 1,125 km s^−1^ for both tunings. There was also an emission feature at the velocity of 1,125 km s^−1^ for the SiO *J*=2−1 (*υ*=1) line (see Supplementary Fig. 3) at ~3.5 *σ* level, whereas no signal was detected at a similar velocity for SiO *J*=2−1 (*υ*=2). On the basis of the detection results at different frequency tunings and the emission of the *υ*=1 line, we posit that the emission of SiO *J*=2−1 (*υ*=3) at the velocity of 1,125 km s^−1^ was a real emission from NGC 1068. Another emission feature of the SiO *J*=2−1 (*υ*=3) line was detected at the velocity of 1,362 km s^−1^, with a slightly weaker peak brightness temperature but a larger velocity-integrated flux than that at the velocity of 1,125 km s^−1^; the counterpart of SiO *J*=2−1 (*υ*=1) was also detected at the similar velocity (see Supplementary Fig. 3).

This source was located at a galactic latitude of −51.9° at which it would be almost impossible to receive strong molecular emissions from the Milky Way. The line survey toward Sgr B2 and Orion-KL^34^ shows that the only possible assignment for the SiO *J*=2−1 (*υ*=3) at velocities of 1,125 and 1,362 km s^−1^ were *c*-C_3_H_2_ at the frequency of 84.727691 GHz with velocities of 26.7 and 264.1 km s^−1^. However, the absence of *c*-C_3_H_2_ emission at the frequency of 85.338906 GHz with similar velocity, which is approximately three times stronger than the emission at the frequency of 84.727691 GHz in Sgr B2 and Orion-KL^34^, means that such emissions could not have originated from a Milky Way source.

### Estimation of isotropic luminosities of SiO and CH_3_OH mega-masers

The isotropic luminosities were calculated by multiplying the flux densities (in units of Jy km s^−1^) by the squares of the distances from the sources. We calculated the isotropic luminosity of the SiO *J*=2−1 (*υ*=3) emissions in *χ* Cyg, to be 0.3 K km s^−1^ × 54 Jy K^−1^ × 0.11^2^ kpc^2^=0.196 Jy km s^−1^ kpc^2^, using a conversion factor of 1 K≃54 Jy at 86 GHz (ref. 17). Our detections of SiO *J*=2−1 (*υ*=3) were 8.86±1.76 mK km s^−1^ at 1,125 km s^−1^ using a single component Gaussian fit, and 29.4±2.7 mK km s^−1^ at 1,362 km s^−1^, by integrating the velocity using the task ‘print area’ in CLASS from 1,357 to 1,365 km s^−1^. Using a conversion factor of 4.2 Jy K^−1^ for the IRAM 30-m at 86 GHz and a distance of 14.4 Mpc (ref. 30), we obtained (8.86±1.76) × 4.2 × 14.4^2^ × 10^3^=(7.7±1.5) × 10^6^ Jy km s^−1^ kpc^2^ for the 1,125 km s^−1^ component and (29.4±2.7) × 4.2 × 14.4^2^ × 10^3^=(2.6±0.24) × 10^7^ Jy km s^−1^ kpc^2^ for the 1,362 km s^−1^ component. Using the same conversion factor, we obtained an isotropic luminosity of CH_3_OH of (1.1±0.2) × 10^8^ Jy km s^−1^ kpc^2^ at 125.1±24.7 mK km s^−1^ using a single component Gaussian fitting and we used a distance from Orion of 414 pc (ref. 35) to obtain an isotropic luminosity of CH_3_OH in the OMC-2 of 34.3 Jy km s^−1^ kpc^2^.

### Number of SiO mega-masers in local type II AGN available using ALMA

Local Seyferts and LINERs were analysed using the CfA Redshift Survey catalogue, as a magnitude-limited sample with 82 sources^36^. Using the catalogue of galaxies that is classified on the CfA Redshift Survey website ( https://www.cfa.harvard.edu/ dfabricant/huchra/zcat/), we found 110 Seyfert II galaxies with velocities below 7,300 km s^−1^, which correspond to ~100 Mpc and can be revealed to 1 pc (2 milliarcseconds) using the ALMA. The sources in the CfA Redshift Survey were with *δ*>−2.5° and the ALMA can be used to observe sources with at least *δ*<+25°; thus, we can expect that over 150 Seyfert II galaxies within 100 Mpc could be observed using the ALMA. Including other type II AGNs, over 20 sources could be available to estimate the masses of SMBHs using the ALMA observations of SiO masers for the fraction of ~10%.

## Author contributions

J.W. wrote the text, obtained and reduced the data, and led the initial observing proposal. J.Z., Y.G. and Z.-Y.Z. were involved in the initial observing proposal and helped to improve the text. D.L., M.F. and Y.S. helped to improve the text. J.Z. and Z.-Y.Z. also assisted with the analysis of the data.

## Additional information

**How to cite this article**: Wang, J. *et al.* SiO and CH_3_OH mega-masers in NGC 1068. *Nat. Commun.* 5:5449 doi: 10.1038/ncomms6449 (2014).

## Supplementary Material

Supplementary Information Supplementary Figures 1-3

## References

[b1] Elitzur M. Astronomical masers. Annu. Rev. Astron. Astrophys. 30, 75–122 (1992).

[b2] Phillips R. B. *et al.* Coordinated Millimeter VLBI Array observations of R Cassiopeiae: 86 GHz SiO masers and envelope dynamics. Astron. J. 122, 2679–2685 (2001).

[b3] van Loon J. T. *et al.* Discovery of the first extragalactic SiO maser. Astron. Astrophys. 306, L29–L32 (1996).

[b4] Cragg D. M. *et al.* Pumping the interstellar methanol masers. Mon. Not. R. Astron. Soc. 259, 203–208 (1992).

[b5] Dos Santos P. M. & Lepine J. R. D. Detection of strong H_2_O emission from galaxy NGC4945. Nature 278, 34–35 (1979).

[b6] Baan W. A. *et al.* Broad hydroxyl emission in IC 4553. Astrophys. J. Lett. 260, 49–52 (1982).

[b7] Baan W. A. *et al.* Formaldehyde absorption and maser emission in galaxies. Astrophys. J. 305, 830–836 (1986).

[b8] Lo K. Y. Mega-masers and galaxies. Annu. Rev. Astron. Astrophys. 43, 625–676 (2005).

[b9] Braatz J. A. *et al.* The Megamaser Cosmology Project. II. The Angular-diameter distance to UGC 3789. Astrophys. J. 718, 657–665 (2010).

[b10] Darling J. & Giovanelli R. A search for OH megamasers at *z*>0.1. III. The complete survey. Astrophys. J. 124, 100–126 (2002).

[b11] Ellingsen S. P. *et al.* A Search for extragalactic methanol masers. Mon. Not. R. Astron. Soc. 267, 510–512 (1994).

[b12] Phillips C. J. *et al.* A comprehensive search for extragalactic 6.7-GHz methanol masers. Mon. Not. R. Astron. Soc. 267, 510–512 (1994).

[b13] Darling J. *et al.* A search for 6.7 GHz methanol masers in OH megamaser galaxies at 0.11< *z* <0.27. Astron. J. 125, 1177–1181 (2003).

[b14] Ellingsen S. P. *et al.* Detection of 36 GHz Class I methanol maser emission towards NGC 253. Astrophys. J. Lett. 790, 28–32 (2014).

[b15] Schinnerer E. *et al.* Bars and warps traced by the molecular gas in the Seyfert 2 galaxy NGC 1068. Astrophys. J. 533, 850–868 (2000).

[b16] Garcia-Burillo S. *et al.* Molecular line emission in NGC1068 imaged with ALMA. I An AGN-driven outflow in the dense molecular gas. Astron. Astrophys. 567, A125 (2014).

[b17] Cho S. *et al.* First detection of the SiO (*v*=3, *J*=2−1) maser emission from χ Cygni. Astrophys. J. 657, 482–485 (2007).

[b18] Batrla W. & Menten K. M. Detection of a strong new maser line of methanol toward DR 21(OH). Astrophys. J. Lett. 329, 117–120 (1988).

[b19] Menten K. M. *et al.* Methanol in the Orion region. I - Millimeter-wave observations. II - The 25 GHz masers revisited. Astron. Astrophys. 198, 253–273 (1988).

[b20] Sutton A. M. *et al.* Methanol in W3(H_2_O) and surrounding regions. Astrophys. J. 609, 231–242 (2004).

[b21] Takano S. *et al.* Distributions of molecules in the circumnuclear disk and surrounding starburst ring in the Seyfert galaxy NGC 1068 observed with ALMA. Publ. Astron. Soc. Jpn 66, 75–88 (2014).

[b22] Ramstedt S. *et al.* Circumstellar molecular line emission from S-type AGB stars: mass-loss rates and SiO abundances. Astron. Astrophys. 499, 515–527 (2009).

[b23] Wiesemeyer H., Thum C. & Walmsley C. M. The polarization of mm methanol masers. Astron. Astrophys. 428, 479–495 (2004).

[b24] Garca-Burillo S *et al.* Molecular gas chemistry in AGN. II. High-resolution imaging of SiO emission in NGC 1068: shocks or XDR? Astron. Astrophys. 519, 2–18 (2010).

[b25] Sinclair M. W. *et al.* A methanol maser in the Large Magellanic Cloud. Mon. Not. R. Astron. Soc. 256, 33–34 (1992).

[b26] Beasley A. J. *et al.* A methanol maser survey of IRAS-selected regions in the Magellanic Clouds. Astrophys. J. 459, 600–605 (1996).

[b27] Green J. A. *et al.* Multibeam maser survey of methanol and excited OH in the Magellanic Clouds: new detections and maser abundance estimates. Mon. Not. R. Astron. Soc. 385, 948–956 (2008).

[b28] Ellingsen S. P. *et al.* Masers associated with high-mass star formation regions in the Large Magellanic Cloud. Mon. Not. R. Astron. Soc. 404, 779–791 (2010).

[b29] Sjouwerman L. O. *et al.* Discovery of the first methanol (CH_3_OH) maser in the Andromeda Galaxy (M 31). Astrophys. J. 724, 158–160 (2010).

[b30] Gallimore J. F. *et al.* The nature of the nuclear H_2_O masers of NGC1068: reverberation and evidence for a rotating disk geometry. Astrophys. J. 556, 694–715 (2001).

[b31] Kuo C. Y. *et al.* The Mega-maser Cosmology Project. III. accurate masses of seven supermassive black holes in active galaxies with circumnuclear mega-maser disks. Astrophys. J. 727, 20–34 (2011).

[b32] Usero A., Garca-Burillo S., Fuente A., Martn-Pintado J. & Rodrguez-Fernández N. J. Molecular gas chemistry in AGN. I. The IRAM 30 m survey of NGC 1068. Astron. Astrophys. 419, 897–912 (2004).

[b33] Aladro R. *et al.* A λ=3 mm molecular line survey of NGC 1068. Chemical signatures of an AGN environment. Astron. Astrophys. 549, A39 (2013).

[b34] Turner B. E. A molecular line survey of Sagittarius B2 and Orion-KL from 70 to 115 GHz. I - The observational data. Astrophys. J. Supp. 70, 539–622 (1989).

[b35] Menten K. M. *et al.* The distance to the Orion Nebula. Astron. Astrophys. 474, 515–520 (2007).

[b36] Huchra J. & Burg R. The spatial distribution of active galactic nuclei. I - The density of Seyfert galaxies and liners. Astrophys. J. 393, 90–97 (1992).

