# Supplementary material for: SiO and CH3OH mega-masers in NGC 1068
Source: Nat Commun. 2014 Nov 11;5:5449. doi: 10.1038/ncomms6449 (PMC4241987; doi:10.1038/ncomms6449)
Supplement: Supplementary Information — Supplementary Figures 1-3 [file ncomms6449-s1.pdf]

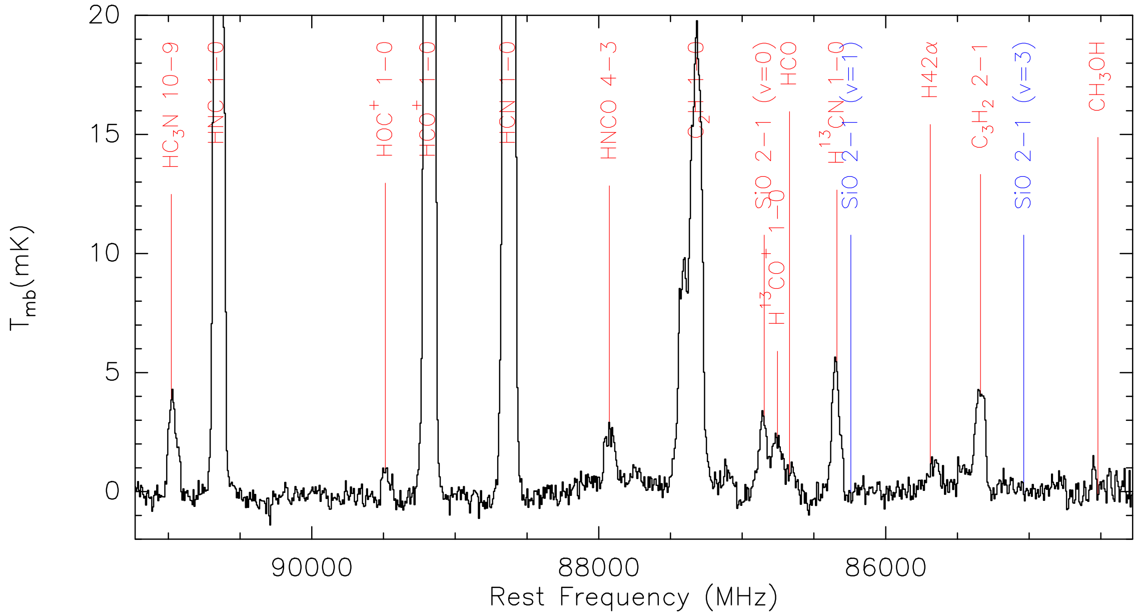

Supplementary Figure 1. 8 GHz bandwidth spectrum at 3mm band of NGC 1068 obtained by IRAM 30m, with the frequency resolution of about 7 MHz, which corresponds to $\sim$ 24.3 km s^{-1} at 86.8GHz. The X axis is the frequency referred to the radio defined velocity of 1137 km s^{-1}, while Y axis is the main beam brightness temperature.

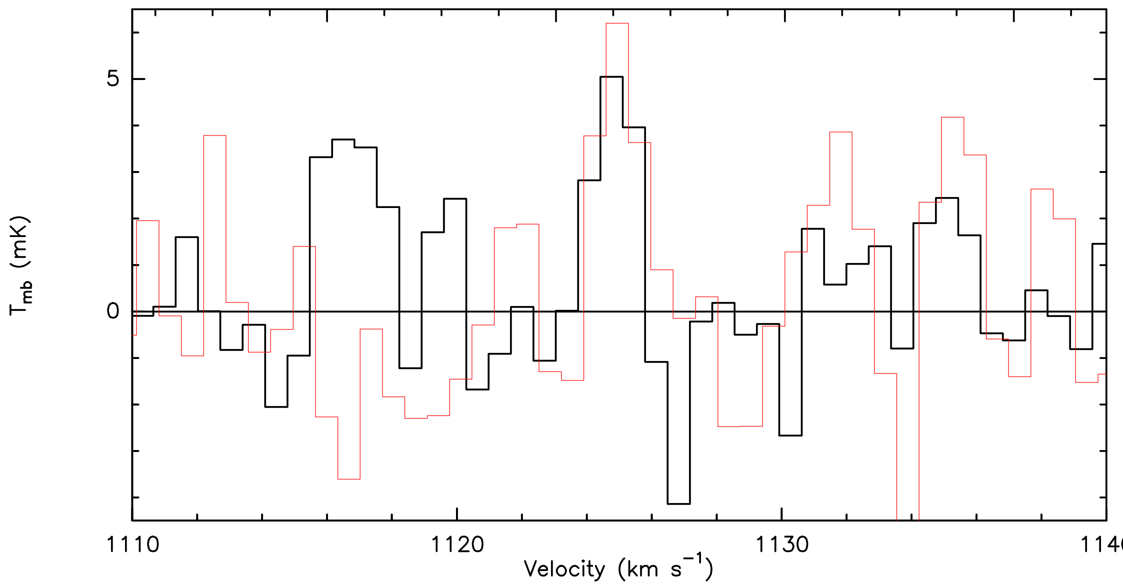

Supplementary Figure 2. SiO J=2-1 (v = 3) lines in NGC 1068 detected by IRAM 30m telescope with different center frequency tuning. The black line is with the $H^{13}CO^+$ 1-0 (86.754288 GHz) at the center of Lower Outer part of EMIR, while the red one is for that with $H^{13}CN$ 1-0 (86.340176 GHz) at the same place.

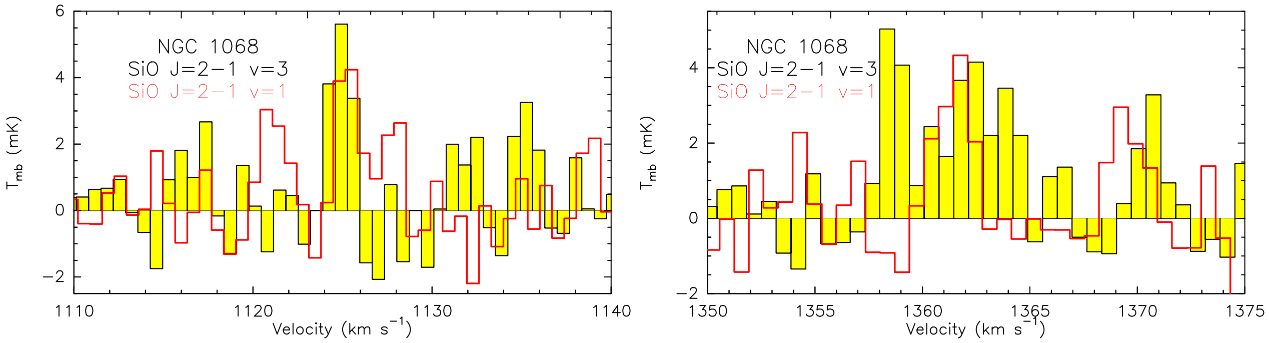

Supplementary Figure 3. SiO J=2-1 (v = 3) lines (black line) in NGC 1068 overlaid by SiO J=2-1 (v = 1) line (red line) at the velocity of 1125 km s^{-1} (*left*) and 1362 km s^{-1} (*right*).
